# Eliminating malaria in Malaysia: the role of partnerships between the public and commercial sectors in Sabah

**DOI:** 10.1186/1475-2875-13-24

**Published:** 2014-01-21

**Authors:** Kelly C Sanders, Christina Rundi, Jenarun Jelip, Yusof Rashman, Cara Smith Gueye, Roly D Gosling

**Affiliations:** 1 Global Health Group, University of California, San Francisco, 50 Beale Street, Suite 1200, San Francisco, CA, USA; 2 Sabah State Health Department, Ministry of Health, Malaysia, 3rd Floor, Federal Buildings, 88814 Kota Kinabalu, Sabah, Malaysia; 3 Sabah Vector Borne Disease Control Programme, Ministry of Health, Malaysia, 1st Floor, Federal Buildings, PO Box 11290, 88814 Kota Kinabalu, Sabah, Malaysia; 4 Vector Borne Disease Sector, Disease Control Division, Ministry of Health, Malaysia, Level 4, Block E10, Complex E, Central Federal Government Administration, 62590 Putrajaya, Malaysia

**Keywords:** Malaria, Malaria elimination, Public-private partnerships, Plantations, Malaysia, Sabah, Industry, Commercial sector

## Abstract

**Background:**

Countries in the Asia Pacific region have made great progress in the fight against malaria; several are rapidly approaching elimination. However, malaria control programmes operating in elimination settings face substantial challenges, particularly around mobile migrant populations, access to remote areas and the diversity of vectors with varying biting and breeding behaviours. These challenges can be addressed through subnational collaborations with commercial partners, such as mining or plantation companies, that can conduct or support malaria control activities to cover employees. Such partnerships can be a useful tool for accessing high-risk populations and supporting malaria elimination goals.

**Methods:**

This observational qualitative case study employed semi-structured key informant interviews to describe partnerships between the Malaysian Malaria Control Programme (MCP), and private palm oil, rubber and acacia plantations in the state of Sabah. Semi-structured interview guides were used to examine resource commitments, incentives, challenges, and successes of the collaborations.

**Results:**

Interviews with workers from private plantations and the state of Sabah MCP indicated that partnerships with the commercial sector had contributed to decreases in incidence at plantation sites since 1991. Several plantations contribute financial and human resources toward malaria control efforts and all plantations frequently communicate with the MCP to help monitor the malaria situation on-site. Management of partnerships between private corporations and government entities can be challenging, as prioritization of malaria control may change with annual profits or arrival of new management.

**Conclusions:**

Partnering with the commercial sector has been an essential operational strategy to support malaria elimination in Sabah. The successes of these partnerships rely on a common understanding that elimination will be a mutually beneficial outcome for employers and the general public. Best practices included consistent communication, developing government-staffed subsector offices for malaria control on-site, engaging commercial plantations to provide financial and human resources for malaria control activities, and the development of new worker screening programmes. The successes and challenges associated with partnerships between the public and commercial sector can serve as an example for other malaria-eliminating countries with large plantation sectors, and may also be applied to other sectors that employ migrant workers or have commercial enterprises in hard to reach areas.

## Background

Malaria continues to cause major morbidity in the Asia Pacific region, with approximately 2.2 billion people at risk for the disease [1]. The malaria-endemic countries in this region account for almost 30 million malaria cases, representing 84% of global malaria cases outside Africa [2]. Despite major operational challenges and the persistence of receptive areas that are conducive to malaria transmission, many countries have made major strides in decreasing malaria incidence and morbidity over the last ten years [2-4]. Malaria elimination, defined as the interruption of local malaria transmission in a specific geographical area [3], is gaining momentum in the region. The Republic of Korea, Malaysia and Sri Lanka are quickly approaching elimination, having committed to national malaria elimination goals by the year 2020 [2].

Malaria control programmes face substantial challenges on the path towards elimination, including the high diversity of vectors and vector behaviour in the Asia Pacific region [4] and the large mobile populations that move between countries of varying malaria risk. These populations are often located in remote areas and are difficult to target and access for surveillance and vector control [5]. Additionally, reductions in financing for malaria programmes are a barrier to success; maintaining financing is crucial to preserve quality and coverage of interventions as countries near elimination.

Under certain conditions, it has been shown that agricultural, plantation, mining, and timber extraction activities have caused increases in malaria incidence and contributed to malaria resurgences [6-11]. These industries often hire workers from around the region, increasing the risk of importation. One way to meet these collective challenges is through collaborative efforts with the private sector [12-14].

Malaria control programmes may engage private industry partners, such as manufacturing, mining or plantation companies, to conduct or support malaria case detection and diagnosis, vector control and surveillance activities. Both formal and informal public-private partnerships have been effectively implemented for malaria control worldwide, as well as to control other communicable and non-communicable diseases, and continue to gain political support [12,15,16]. The term “private-public partnerships” has been applied to various types of partnerships at the global, national and local level, including collaborations between global organizations and businesses, partnerships between public and private health sectors, and ground-level commercial business partnerships [12,16]. Much of current research regarding partnerships with the private sector for disease control focuses largely on global collaborations, including research and development partnerships, the development of innovative financing mechanisms and health systems initiatives [12,17-20]. These national and global partnerships, often between governments, non-governmental organizations and large international companies, have been well described and analysed [20-22].

However, small-scale local collaborations between disease control programmes and commercial businesses, specifically aimed at controlling local disease burden, have been less documented. These partnerships often occur at the sub-national level, and may be largely dependent on informal relationships between disease control offices and local businesses [23]. These partnerships face unique challenges. Without formal agreements, both sides rely on goodwill to ensure ongoing commitments to agreed upon responsibilities. Examples of successful commercial partnerships exist from highly endemic areas: in conjunction with the government, the AngloGold Ashanti company in Ghana developed an integrated malaria control programme in 2005, successfully reducing malaria cases in the Obuasi mining community from 6,600 to 1,150 cases a month by 2006 [24]. In 2002, a branch of the Marathon Oil company in Equatorial Guinea identified malaria as a key health issue for employees, and launched a multi-year project to decrease malaria incidence amongst its worker population and nearby villages [24-26]. While these examples, and others like it, provide evidence that disease control programmes are engaging in such partnerships, best practices and challenges are not widely disseminated.

Partnerships between the Malaysian Malaria Control Programme (MCP) and private plantations and agricultural estates have existed in Malaysia since the early 1900s, particularly in the state of Sabah [27,28]. Throughout the 1980s and 90s, Sabah, located on the island of Borneo, experienced high incidence of malaria with ongoing transmission and outbreaks on many plantations. In response, the state operationalized public-private “smart partnerships” with timber extraction companies and palm oil, rubber and acacia plantations, as part of its malaria control strategy [29]. Healthcare is often provided through estate or on-site private clinics at established plantations (though the public health sector provides most care in the country) and the Ministry of Health has provided for antenatal care to pregnant mothers and immunizations to children at many plantations. Through collaborating with private sector partners to conduct malaria control activities, the MCP has worked to address the challenges of ongoing inter- and intra-national migration, remote geography, social, environmental and regulatory issues related to a burgeoning private plantation industry.

This qualitative case study aims to describe the spectrum of informal collaboration between private plantations and the Sabah State MCP, delineating the incentives for collaboration from the perspective of the government and private companies, and the financial, human, material and logistical inputs provided by each group. The study illustrates how optimizing partnerships between national malaria control programmes and the private sector can help control malaria and contribute to elimination.

## Methods

### Document review

Published and unpublished grey literature was identified and reviewed to inform interview guides and as background for the case study. Document searches were conducted using Pubmed, Google and Google Scholar, the World Health Organization archives, WorldCat, and the Yale Harvey Cushing/John Hay Whitney Historical Medical Library. Search terms included: public-private partnerships for health, malaria in Malaysia/Sabah, malaria on plantations, health on palm oil plantations/acacia plantations/rubber plantations, collaborations between public and commercial sectors, immigration in Malaysia/Sabah, disease control partnerships, success factors for public-private partnerships in health, partnerships for disease control, migration and malaria, plantations in Southeast Asia, Business-Public Health partnerships and incentives for commercial sector health partnerships. Reference lists of identified articles were searched to find other relevant studies.

Document reviews to provide additional background information for the case study were conducted in country at the Malaysian Ministry of Health Malaria Control Programme office, the Sabah State Malaria Control Programme office, the Sabah State Public Library, and the Institute of Medical Research in Kuala Lumpur. Grey literature and annual reports, administrative reports, action plans and data from government databases were also obtained from these sources.

### Design

This study was an observational qualitative study. In addition to the document review, semi-structured key informant interviews were conducted in 2012 to describe collaborations between the Sabah State MCP and private plantations [30,31]. Interviews were conducted in five districts in Sabah State, where the private plantation and MCP collaborations have been used as an operational strategy to decrease malaria incidence. Semi-structured interview guides were developed in collaboration with the Malaysian MCP at the state and national level and included questions that focused on description of the current collaboration, including types and frequency of communication between MCP and plantation staff, development of the collaboration, resource commitment, activities conducted by the MCP and plantation staff or management, perception of challenges and successes of the partnership, discussion of incentives for partnering and migration. Data on malaria cases were obtained from the Sabah MCP.

### Sampling and participants

A purposive sampling method was used for the interview portion of this case study [32]. Seven private plantations were included, as well as the state MCP office and four district level MCP offices. Plantations were chosen based on the recommendation of the Sabah MCP, informed by three criteria: (1) reported a high number of malaria cases in the last 20 years or experienced an outbreak or malaria death on-site; (2) were engaged in a functioning partnership with the MCP; and, (3) were accessible by vehicle. Interviews with informants lasted between 45 minutes to three hours.

Plantation informants were identified by MCP staff based on involvement in existing collaborations with plantations. These included operations managers, health and safety officers, human resource officers, hospital assistants, quality assurance officers and plantation health personnel. Out of seven plantations identified as targets for the case study, six were able to provide personnel to participate in interviews. Demographic details regarding sampled plantations are provided in Table 1. Participants from the Sabah MCP included assistant environmental health officers of various levels, entomologists, spraymen, and several top managers in the Programme. MCP officers were chosen for interviews based on their knowledge of malaria and their current or past involvement working with private plantations.

**Table 1:** Demographic information for sampled plantations

**Plantation**	**Year partnership started**	**Plantation type**	**Current size of workforce (Approximate)**	Malaria cases, annual*
**Plantation I**	2009	Rubber, Timber Extraction	600	2009: 35 case outbreak in 1 month
				2010: 4 cases
				2011: 6 cases
**Plantation II**	Early 2000s	Palm Oil, Rubber	518	2001: Estimates of 1000+
				2010: 3 cases
				2011: 3 cases
**Plantation III**	2009	Palm Oil, Oil Manufacturing Plant	439	2009: 300 cases
				2010: 7 cases at plant on-site
				2011: 3 cases at plant on-site
**Plantation IV**	1990s	Palm Oil	952	1990s: Estimates of 400+
				2010: 0 cases
				2011: 0 cases
**Plantation V**	2004	Palm Oil	114	2005: 11 cases
				2010: 6 cases
				2011: 3 cases
**Plantation VI**	2000	Palm Oil	11,231	2000: Estimates of 200+
				2010: 6 cases
				2011: 4 cases
**Plantation VII**	Since 1978; New collaboration since privatization in 2007	Acacia, Paper Manufacturing	Current workforce unknown by management: Goal of 4,000 workers in next 5 years	1978: High, exact number unknown, outbreaks common
				2010: 5 cases
				2011: 1 cases

*Includes annual case load at commencement of partnership and cases in 2010 & 2011 for each plantation; multiple plantations may be under the jurisdiction of one district office.

Informant responses were separated by themes, which were then examined for similarities and differences across plantation and MCP informants. The description of the context/setting and findings according to these major themes were assembled from document review, programme data and the interviews. These findings were triangulated across sources of data.

### Analysis

Interviews were conducted in English, with translation from the Malay language to English by MCP officers in the rare occasion that an informant was unable to respond in English. Interviews were digitally recorded, or notes were taken by hand if preferred by the informant. All notes were transcribed and coded line-by-line to identify emergent themes [25]. Analysis of individual and group interview transcripts was conducted in the qualitative coding software ATLAS.ti (version 6.1).

### Ethical considerations

The researchers sought and received ethical approval from the University of California, San Francisco Committee on Human Research, the Malaysian National Institute of Health (NIH) Institute for Health Behavioural Research (IHBR), and the Malaysian National Medical Research Register. The Malaysian Ministry of Health also approved the study. Informed written consent for qualitative interviews was obtained from all study participants.

## Results

### Epidemiology of malaria in Malaysia and the state of Sabah

**Figure 1:**
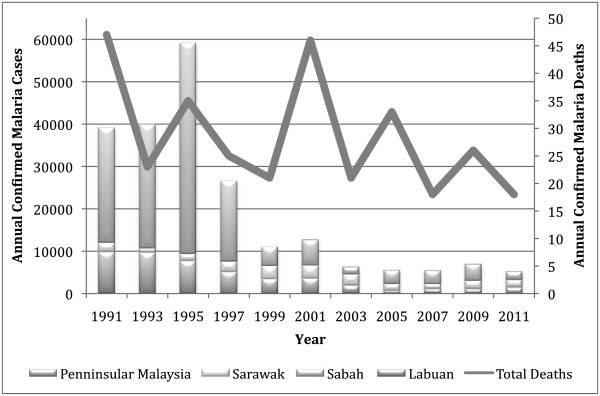
**Confirmed malaria cases and deaths in Malaysia **[33]**.**

Malaria remains a substantial contributor to morbidity in Malaysia. Malaria epidemiology is varied across the country, largely due to diverse ecologic conditions and vectors. Peninsular Malaysia, a conglomerate of 11 states and two federal territories, experienced rapid declines in malaria incidence in the 1970s and 1980s [27]. Cases in Peninsular Malaysia continued to decline throughout the 1990s, from approximately 10,000 in 1994 to 1,512 in 2011, with a majority of cases imported from neighbouring endemic countries [33]. The Sarawak State, located on Borneo, has maintained between 1,000 and 3,000 cases annually since the early 1990s, while the Sabah State (also on Borneo) has experienced the most dramatic decreases in incidence from 49,192 cases in 1994 to 2,032 cases in 2011 (Figure 1) [33]. Cases in Sabah are predominantly *Plasmodium falciparum* and *Plasmodium vivax.*

In light of the differing logistical and technical challenges in each region of the country, Malaysia has a geographically phased malaria elimination goal of zero local transmission for West Malaysia by 2015, and 2020 for Sabah and Sarawak. Since 1994, the national level MCP, located in Peninsular Malaysia, has devoted increased resources to malaria control in Sabah, acknowledging the logistical and geographic challenges of the state MCP. Mountainous, remote geography, and a lack of infrastructure with isolated communities challenge effective control measures. Due to a burgeoning palm oil and rubber plantation sector, Sabah also employs a large number of foreign workers from Indonesia and the Philippines, two nearby endemic countries. Cases amongst foreign nationals from Indonesia and Philippines have ranged from 30% to 45% of total annual cases in Sabah since 2007. In recent years, the state implemented stricter mandatory screening laws, and the proportion of cases began to decrease amongst migrants (Table 2).

**Table 2:** **Cases by nationality in Sabah, Malaysia, 2008–2010 **[33]

**Nationality**	**2010**		**2009**		**2008**	
	**Number of cases**	**% of total cases**	**Number of cases**	**% of total cases**	**Number of cases**	**% of total cases**
**Malaysia**	1,741	65.8%	2,428	60.6%	2400	58.0%
**Indonesia**	541	20.5%	1,144	28.5%	1317	31.9%
**Philippines**	349	13.2%	431	10.8%	418	10.1%
**Other foreign nationals**	13	0.5%	6	0.1%	0	0.0%

### Plantations in Southeast Asia and Malaysia

The climate in Southeast Asia supports large-scale production of a variety of products grown on large estates and plantations, namely rubber, palm oil, acacia (for paper), and coffee; today millions of hectares of land are devoted to plantation monocrops [7,8,34,35]. Global demand for palm oil, in particular, continues to grow and the industry employs hundreds of thousands of local and migrant workers across the region [33,36].

A particularly robust plantation industry exists in Malaysia. During British colonial times, coffee and rubber plantations were highly profitable, though largely concentrated in Peninsular Malaysia [27]. Over the last century, the Malaysian government has prioritized land development for plantation crops and the plantation industry in the state of Sabah on Borneo has grown rapidly. As one of the highest yielding oil crops, palm oil production has increased in Sabah in recent years, resulting in forest being cleared for planting in increasingly remote areas [35]. Private corporations, both domestic and international, manage operations, with many owning multiple plantations across the country. Plantations in Sabah are often located on government land and companies are granted a 99-year lease. Larger plantations may consist of multiple estates in a geographic area, with a separate local management structure for each, and typically recruit workers from nearby Indonesia and the Philippines. The terms ‘estate’ and ‘plantation’ are often used synonymously, and some plantations consist of multiple estates, which may be under different local management. While the exact number of plantations in Sabah is unavailable, roughly 1,077 estates across the state are currently under surveillance by the MCP [37]. This number is an underestimate, however, as small-holder plantations, consisting of fewer than 50 employees, are not required to register with the government. Additionally, the state MCP only recently (2010) began to formally collect and disaggregate data related to annual cases occurring on plantations, though local district offices have monitored cases on plantations for some time. In 2010, 277 cases were reported from plantations across the state; 107 cases were reported in 2011 [37].

Collected data and qualitative interviews indicated that all plantations have experienced a decrease in malaria incidence since partnering with the government (Table 1), although outside environmental and social factors may have also contributed to these reductions. At the time each relationship was developed (varying from 1991 to 2011), cases reported in the plantations ranged widely, from 11 to over 1,000 annually.

### Development of partnerships

Partnerships between plantations and the MCP documented in this case study were typically developed for one of two reasons: (1) high levels of ongoing malaria transmission; or (2) occurrence of an outbreak or death on-site. In each case, the state or district MCP offices contacted plantation management to discuss the possibility of partnering as a strategy to address the local malaria situation. Although plantation management and clinic employees were generally supportive of developing a malaria control partnership with the government, agreement on partnership structure, including the division of roles and responsibilities for each group, on average took about one year to establish.

District MCP participants noted that in the last year, two non-sampled plantations have contacted the MCP to discuss the potential of partnering, possibly indicating that benefits of these public-private partnerships are becoming more widely understood and discussed within the plantation industry in Sabah.

### Incentives for participating in partnerships

The Sabah MCP indicated three reasons for collaborating with the commercial sector: (1) plantations are often located in remote geographic areas that are challenging to access and are too time intensive to reach consistently; (2) plantations often recruit immigrants, both documented and undocumented, who are at higher risk for malaria for multiple reasons; and (3) collaborations in which plantation management provide financial or human resources to control malaria free up critical MCP resources to focus on local communities with ongoing transmission.

MCP staff face significant barriers to accessing populations that work on remote plantations. Partnering with plantations allows expanded coverage of vulnerable populations by malaria control interventions. Plantation workers are typically foreign migrants arriving from endemic areas of the Philippines and Indonesia, and may import malaria from their country of origin to Sabah (Table 2). Although the MCP aims to cover all at risk populations with vector control [indoor residual spraying (IRS) and insecticide treated nets (ITNs) and surveillance activities (mass blood surveys (MBS)], it does not have the resources required for 100% coverage of these measures. Plantation workers are disbursed across wide geographic distances and work at variable hours, including overnight. It is especially challenging to provide malaria control for new developing plantations, as workers often clear land in highly remote and forested areas, and typically work at night and into the early morning. Additionally, migrants are also often highly mobile, working for short times in one plantation and then moving onto another, posing challenges to appropriate case follow-up by the MCP.

Developing robust partnerships with the government helps ensure that migrants are provided with adequate health care, either on-site by plantation clinics, or through access to government services. Undocumented migrant workers, particularly those living on plantations without private clinics, may avoid accessing health care for suspected malaria out of fear of deportation by immigration authorities, or may lack education regarding malaria and related symptoms. MCP officers and plantation managers felt that consistent interactions with MCP officers helped build trust within the migrant community, spurring workers to quickly alert plantation staff or MCP officers when they fall ill.

Plantation managers reported different, though complementary, incentives to collaborate. Those most frequently mentioned included: (1) worker productivity (Plantations I, II, III, IV, V, VII); (2) social responsibility (Plantations I, V, VII); (3) attracting workers (Plantations I, II); (4) decreasing employee health costs (Plantations I, IV); and (5) abiding by labour law (elaborated below) (Plantations I, IV).

The most frequent reason cited by plantation managers for partnering with the MCP was ensuring a high level of worker productivity by decreasing the impact of malaria on-site. Although managers did not mention any specific analyses conducted on the effects of absenteeism on profit due to malaria, they did indicate that workers who are unable to work directly impact economic productivity of the plantation. Concerns around absenteeism were particularly emphasized by managers at Plantation I, a new plantation rapidly growing in size and economic output. While most sampled plantations currently have low endemicity, managers indicated concerns about the potential negative impact on economic productivity that would occur should an outbreak occur on site.

Decreasing costs associated with transporting cases to government clinics or hospitals, particularly for plantations without a private on-site clinic, was also mentioned as an incentive to partner with the MCP. Management at Plantation II estimated a cost of approximately 200 MYR, or 65 USD, for one-way transport to a clinic. This was seen as an avoidable cost, and cause for concern should an outbreak occur on-site.

Abiding by Malaysian labour law and policies was also mentioned as an incentive to collaborate. Worker labour law requires employers to provide health care, either in the private or public sector, for workers in addition to ensuring a healthy workplace. Although this legal framework does not currently contain language requiring private industry to specifically support or conduct malaria control measures as a preventative health measure, two plantations, I and IV, felt that the MCP collaborations fell within the general worker welfare requirements mandated by the government.

Although more challenging to quantify, ‘social responsibility’ was noted as an important motivation to collaborate with the government. Several plantation managers noted that collaboration with the MCP was an example of how the commercial sector could contribute to the general health of Malaysian society, and was part of their company’s policy of corporate social responsibility.

### Division of labour and resources

There is a diverse array of resource commitments and divisions of labour between district MCP offices and plantations for these collaborations. The MCP provides more or less support depending on the willingness and ability of plantations to fund malaria control interventions.

Plantations I, II and III currently rely on the control programme to conduct IRS and ITN retreatment. Malaysian national malaria control policy calls for twice-yearly ITN distribution/retreatment and IRS in endemic areas, with immediate coverage by both control measures in the event of an outbreak (Table 3).

**Table 3:** Contributions to IRS/ITN re/treatment and distribution by individual plantations and the Malaria Control Programme (MCP)

**Plantation collaboration**	**I**	**II**	**III**	**IV**	**V**	**VI**	**VII**
**IRS/ITN re/treatment**	PL in negotiation to cover; currently covered by MCP	PL in negotiation to cover; currently covered by MCP	PL in negotiation to cover; currently covered by MCP	PL subcontracts out; MCP supervises activities	PL conducts, MCP supervises activities	PL subcontracts out; MCP supervises activities	MCP covers
**ITN distribution**	PL in negotiation; currently covered by MCP during outbreaks	50% of workers currently covered by PL, MCP covers during outbreaks	MCP covers during outbreaks	MCP covers during outbreaks	PL covers workers; MCP may add during outbreaks	PL distributes on ad-hoc basis; MCP covers during outbreaks	MCP covers during outbreaks

MCP (Malaria Control Programme) in this table refers to the Malaria Control Programme District Office that works with each plantation. Some MCP district offices have jurisdiction over multiple plantations included in the case study. PL refers to the Plantation involved in a partnership with the MCP.

In large districts with remote populations, malaria control officers struggle to meet deadlines for bi-annual IRS coverage because of the time required to travel to rural areas. Malaria control officers noted that the commitment of Plantations IV, V, and VI to conducting IRS and ITN distribution and retreatment has been critical to achieving coverage goals. As noted in Table 3, the MCP and Plantations I, II and III are negotiating with the plantations to implement IRS, or provide logistical support, including a vehicle and driver to transport the MCP officers to remote plantation sites for IRS and ITN retreatment. With these arrangements, the MCP aims to either train plantation workers to conduct IRS on their sites, or alternatively the plantation may choose to contract out IRS. Plantations IV, V, VI and VII have chosen this arrangement, paying their workers or a subcontractor to conduct IRS. Plantations indicated that IRS is costly due to the size and remoteness of their sites, but managers of Plantations IV and V stated that these costs were an investment in the health of their workers, and would prevent outbreaks from occurring on-site; they plan to continue financing the IRS activities.

The MCP closely supervises spraying at plantations that conduct or subcontract IRS; an officer must be on-site with the plantation IRS team for the first few days of spraying activities. This ensures quality IRS, and helps to maintain high coverage, at levels equivalent to those that would occur with an MCP IRS team.

Increasing coverage of ITNs by plantations is currently under discussion at all sites. National policy dictates that the MCP only provide ITNs to Malaysian nationals, with the only exception being in the case of an outbreak. MCP officers urge private plantations to fund and provide ITNs for all workers, regardless of citizenship. Plantations II and IV currently provide ITNs for the majority of workers. Plantation I is evaluating the need for ITN distribution on-site as management noted that, during discussions with workers, workers indicated that they did not want ITNs. Another barrier is the cost of ITNs, especially for Plantation I and VII that are planning major increases in the number of workers over the next five years. The mobility of plantation workers, who often take ITNs provided to them by management when they move to other plantations for employment, is also a major concern to plantation management and the MCP. Plantations are reluctant to devote considerable resources for ITNs if they believe they are likely to lose that investment.

Because plantations often hire migrants from nearby endemic countries, prevention of malaria importation is crucial. Every plantation, with the exception of Plantation VII, has mandatory screening of workers upon arrival at the plantation ‘base camp’. As a rule, every new worker must check in before beginning work, and management calls the MCP to conduct screening for malaria (Table 4). However, waiting for the MCP to reach remote plantations can cause delays of several days for malaria screening, and several plantations (I, II, III and V) now have trained volunteers from management (Table 4) to screen new workers. Interviews also revealed that parent corporations may use subcontractors to hire temporary workers, both documented and undocumented, who frequently skip the initial plantation registration process. The MCP is working with all sampled plantations to identify ways to access and promptly screen these groups, and is looking for ways to collaborate with new plantations on similar activities.

**Table 4:** Plantation and Malaria Control Programme screening programs and volunteer workers

**Plantation collaboration**	**I**	**II**	**III**	**IV**	**V**	**VI**	**VII**
**Screens new workers**	MCP screens workers	MCP screens workers	MCP screens workers	No screening	PL screens workers	No screening	No screening
**Utilizes volunteer workers**	PL uses volunteers, MCP trains	PL uses volunteers, MCP trains	PL uses volunteers, MCP trains	No volunteer workers	PL uses volunteers, MCP trains	No volunteer workers	No volunteer workers

MCP (Malaria Control Programme) refers to the district office that works with each plantation, and some MCP district offices have jurisdiction over multiple study plantations; PL refers to the Plantation involved in partnership.

### Surveillance activities

Per national policy, district MCP offices conduct all surveillance activities. In addition to case investigation, reactive and proactive case detection and bi-annual MBS, the district MCP offices keep detailed records of malaria epidemiology on plantation sites. This data was reported officially to the Sabah State MCP starting in 2007.

### Subsector offices

Two plantations, I and III, have subsector offices, or rural outpost MCP offices, which are designed to reach remote populations with vector control and surveillance (Table 5). These offices cover both plantation sites and nearby communities. While the subsector malaria control officers generally conduct malaria control interventions, some plantations (I, III) provide resources for the offices, including land and office buildings, equipment, electricity and some logistical support. Plantations I and III provide office buildings for the subsector offices, living quarters for officers and transportation for control activities.

**Table 5:** Contributions by plantations and the Malaria Control Programme to subsector offices

**Plantation collaboration**	**I**	**II**	**III**	**IV**	**V**	**VI**	**VII**
**Subsector Office**	PL provides office and housing for staff; MCP provides staffing and equipment	No subsector office	Provided land for office; MCP provides staffing, office and equipment	No subsector office	No subsector office	No subsector office	No subsector office

MCP (Malaria Control Programme) refers to the district office that works with each plantation, and some MCP district offices have jurisdiction over multiple study plantations; PL refers to the Plantation involved in partnership.

The Sabah MCP also trains volunteers to assist subsector MCP officers with malaria control interventions at Plantations I, II, III and V. Volunteers aid in vector control when needed, but more frequently it is the management staff that screen new workers when they arrive at the plantation (Table 5).

### On-site plantation clinics

Four plantations (II, V, VI and VII) have private on-site clinics that are capable of treating malaria cases; Plantations I, VI and VII have microscopes on-site and can diagnose cases. Plantation V is in the process of procuring two microscopes for diagnosis. Clinicians and MCP officers work closely together, with frequent communication. MCP officers noted that clinicians are often able to act as liaisons between patients and plantation management to ensure appropriate treatment of cases and reporting to the MCP.

The MCP is working with other public health programmes in the Sabah Health Department, such as the Family Health Development Programme, to support Plantation I to develop an on-site clinic that will be run by the Sabah State Health Department. The plantation will provide the building, equipment and commodities, while the government will provide staff. The MCP and the management at Plantation I indicated that this partnership illustrates the potential to expand current relationships from malaria control to control of other diseases, involving the state’s overarching public health programme (Table 6).

**Table 6:** Clinics (private or government run) located on plantations

**Plantation collaboration**	**I**	**II**	**III**	**IV**	**V**	**VI**	**VII**
**On-site Clinic**	PL negotiating with Health Department for government clinic on-site	Private clinic on-site	PL negotiating with Health Department for government clinic on-site	No clinic	Private clinic on-site	Government clinic on-site	Private clinic on-site

MCP (Malaria Control Programme) refers to the district office that works with each plantation, and some MCP district offices have jurisdiction over multiple study plantations; PL refers to the Plantation involved in partnership.

### Management of partnerships

Managing collaborations with plantations requires a significant time and resource commitment by the Malaria Control Programme. MCP officers in the plantation subsector offices work with plantation management on a daily basis, discussing vector control strategy, case detection, screening for migrant workers, screenings and health promotion activities. Plantations with clinics also work closely with the MCP; clinic staff typically calls whenever a new worker has been screened or when there is a suspected case. Each plantation, regardless of the level of resource commitment and structure of partnership, is visited each week by district or subsector programme officers, or if that is not possible, at least biannually during IRS and ITN activities. Most communications between partners are informal and ad-hoc, occurring on a daily or weekly basis.

Formal meetings with top management occur during partnership development, or when there is an outbreak. Plantation I, the newest collaboration, has the most significant contact with the MCP. The collaboration began with meetings between the Sabah State MCP and top management at the plantation headquarters, located in the capital city of Sabah, Kota Kinabalu. The formal involvement of the state level MCP was noted as critical to the development of this new partnership.

Another strategy that was noted as successful by both groups was formal recognition of collaboration by awarding certificates of appreciation to plantations that have been particularly cooperative and dedicated to reducing malaria incidence. Plantation managers noted that they were able to utilize these certificates as leverage to incentivize Board members to continue funding for malaria control.

### Challenges in maintaining successful partnerships

Managing partnerships between private corporations and government entities is often a complex task, given the competing priorities, objectives and needs of the different parties. In an effort to ensure ongoing participation, the MCP devotes substantial time to educating plantation management on the importance and benefits of malaria control.

In some cases, the MCP must also dedicate a significant amount of time to supervise partnerships to ensure that plantations conduct the agreed activities. Officers have experienced resistance when trying to access agreed-upon transportation or additional logistical support from plantations. Plantations, on the other hand, find it challenging to provide logistical support when vehicles and workers are needed for plantation work.

Plantation staff turnover can result in a loss of continuity and shift in priorities for the partnership. For example, from 2001–2009, one sampled plantation (II) paid for a full-time team of four workers (three spraymen, one supervisor) to conduct IRS, MBS, and procured ITNs for all workers. However, in 2009, new management discontinued IRS, MBS and ITN distribution because of budgetary constraints, and requested the government to take over these activities, leading to a disruption in control activities. The plantation and MCP are currently discussing transferring responsibility of these activities back to the plantation.

Both sides indicated that the other partner should commit more time and resources to the partnership. Plantation participants believed the government should provide ITNs to all workers, regardless of nationality, and additional malaria control officers for control activities. Several plantation managers requested that the MCP provide formal health education to workers more regularly and frequently.

In contrast, MCP officers working with all plantations in the study (except Plantation VII) felt that additional resources, including ITNs for workers or IRS application, should be the responsibility of the plantation in order to maintain a safe and healthy work environment.

## Discussion

As countries approach malaria elimination targets, collaboration with all stakeholders will be critical to reaching zero cases. Private industry is an untapped resource for malaria control programmes regionally; partners can provide substantial support for malaria control measures and surveillance. Particularly in countries with large migrant worker populations, malaria control programmes will need the support of outside stakeholders to adequately respond to increasing importation risks.

The willingness of some of Sabah’s commercial plantations to engage in conversations around resource allocation, financial commitments, and additional support for preventive measures for malaria elimination is illustrative of the commitment of both partners to maintaining a healthy population, and demonstrates the potential for such partnerships to expand in number, scale and possibly scope by tackling additional public health disease priorities. The successes of existing collaborations can serve as a framework for the process of developing collaborations and serve as a foundation for how to incentivize and structure new partnerships. The lessons learned from these partnerships may also work as a blueprint for similar partnerships with the commercial sector dedicated to malaria elimination in other countries (Table 7).

**Table 7:** Analysis of lessons learned from Sabah MCP experience with partnerships with commercial plantations

	
1)	Use surveillance data to identify plantations with local or imported cases, or those located in high risk areas
2)	Engage plantation staff on-site, or if new relationship, State MCP office engages high level plantation management, on-site or at headquarter offices
3)	Negotiate division of responsibilities and resources with plantation management, preferably with top management to ensure high level commitment; ensure that objectives, expectations and perspectives of both groups are clearly elucidated
4)	Engage plantation staff at all levels (e.g. headquarters, on-site management, subcontractors, workers to educate all staff on the importance of supporting the MCP)
5)	Commit to ongoing and consistent communication between district MCP staff and on-site management and/or clinicians, either daily or weekly depending on need
6)	Large plantations with ongoing cases in risk areas benefit from on-site rural malaria offices
7)	Evaluate areas for improvement on a consistent basis, with frequent MCP staff meetings to assess challenges and identify areas for improvement; consider annual meetings with high level plantation management to assess collaborations, discuss challenges and re-evaluate division of labor and resources based on changes in epidemiology
8)	Formally celebrate plantations that are effectively participating in collaborations, e.g. provide a certificate of recognition

“Best practices” include frequent communication and supervision of activities, either daily or weekly, between plantations and the MCP. This has been critical to problem solving during outbreaks and has allowed for more in-depth monitoring transmission patterns and the epidemiologic situation on-site and in nearby communities. Strong relationships between local MCP staff and plantation management also provide an opportunity to quickly address challenges specifically related to the collaboration. Engagement of top-level management is crucial to ensure buy-in from those making financial decisions in the company, and providing opportunities for both the MCP and commercial partners to evaluate and renegotiate commitments ensures engagement by both parties.

Educating plantation management and subcontractors about the dangers of malaria and the importance of prevention through vector control activities has also been an effective tool for ensuring ongoing resource commitments, particularly when incidence decreases and malaria is no longer viewed by plantation staff as an immediate threat to worker productivity. Similarly, formally acknowledging plantations for their commitment to malaria control has also been useful in solidifying continued participation. For example, one sampled plantation was recently presented an award by the Ministry of Health; it was noted that formal recognition by the MCP was helpful in convincing the company’s Board of Directors on the importance of financial commitments to malaria control.

Building programmes for plantations to screen foreign workers and conducting volunteer trainings for workers to conduct IRS and ITN distribution and retreatment has allowed the MCP to devote resources to decreasing incidence in surrounding villages. Additionally, the development of on-site malaria subsector offices, often jointly supported by plantations, has promoted closer collaboration between the MCP and those sampled plantations with subsector offices. Although subsector offices are resource intensive for the MCP, they allow officers to support activities at plantations that are challenging to reach and provide easier access to nearby rural communities. Table 8 provides an example of potential areas of collaboration for highly functioning partnerships with individual commercial plantations.

**Table 8:** Potential contributions to achieve highly functioning partnerships with commercial sector plantations

**Plantation contributions**	**State and district MCP contributions**
Procure insecticides and conduct IRS (may subcontract to private company or may request training of plantation employees from district level)	Advise plantations on how to procure MCP approved insecticides
Procure, distribute and retreat Insecticide Treated Nets (or distribute Long Lasting Insecticide Treated Nets)	Train and supervise IRS activities
Provide logistical support for MCP officers to assist or supervise 6 monthly IRS/ITN	Supervise ITN distribution; train staff and supervise ITN retreatment
Cover treatment costs of workers who contract malaria, including transportation to health centers (if there is no on-site clinic)	Confirm private clinic malaria diagnosis and supervise treatment of cases, follow up of cases and screening of contacts
Provide on-site clinic for workers, provision of microscopes for detection of malaria by clinic staff	Conduct surveillance - including active and passive case detection
Increase communication (daily or weekly) with MCP regarding potential cases or to alert the MCP of new worker arrival to implement screening	Track local changes in epidemiology (on-site and in nearby communities) and alert plantation staff when outbreaks are detected
Conduct screenings when new workers arrive on-site, or when migrant workers return from endemic home countries	Train volunteers to conduct screenings with RDTs or blood slides
Provide land and/or buildings for on-site subsector offices	Provide trained MCP officer to staff subsector offices

Substantial challenges remain. It is not clear yet how to better incentivize plantations to dedicate financial resources to malaria control when malaria incidence declines. Although the government mandate to support health care for plantation workers was interpreted by two plantations as required participation in malaria prevention and control activities, without a strong legal framework to enforce commitments, the MCP mostly relies on the goodwill of plantations to continue partnerships and adhere to agreements and responsibilities. Interviewees noted two examples of non-sampled plantations that had discontinued IRS campaigns in the last decade as cases decreased to zero. The development of written agreements, or a national legal framework that requires plantations to support malaria control, would support these partnerships in the future.

The plantation industry will likely continue to expand in Malaysia. The large number of plantation workers, both documented and undocumented, needed to support the industry often originate in countries with high endemicity and may import malaria into areas that have reduced or eliminated their malaria burden. These migrant populations can be highly mobile and are typically challenging to reach; they also have increased occupational risk as they are often working outdoors during peak vector biting times [5]. This will continue to pose a substantial threat to effective elimination campaigns in Sabah; working with commercial plantations to ensure that migrant populations are effectively screened and targeted with malaria control measures will decrease the risk of onward transmission for both migrant and local populations [5,38,39]. Finally, smallholder plantations (30–50 employees) will continue to pose a threat to the elimination goal; these plantations are not required to register with the government, and are often challenging to find and access. They are more likely to hire undocumented migrants from endemic countries who are not screened for malaria upon arrival, do not use proper protective measures, and move frequently from place to place. Engaging smallholder plantation owners in malaria control efforts will be critical to moving the elimination agenda forward in Sabah.

### Limitations

While results of the case study show that malaria incidence has declined at all sampled sites since partnerships were developed, direct correlations to reduced cases of malaria cannot be established. Although effectiveness has not been directly quantified, MCP officers and plantation management felt that partnerships play a key role in reducing incidence and prevent further malaria outbreaks in Sabah. Not all plantations with collaborations were sampled, potentially resulting in selection bias. Due to challenges accessing remote areas, the study researcher was accompanied by government MCP officers at each site, which may have led to a degree of social desirability bias. Finally, all interviews were conducted in English, with translation help from staff or malaria officers when necessary, and the nuances of their responses may have been lost in translation.

## Conclusion

The success demonstrated by these seven informal public-private partnerships may serve as examples for other state and district malaria control and elimination programmes both within Malaysia and across the region. Developing a legal framework to support partnerships with the commercial sector may fuel further declines in malaria burden and will provide the structure necessary to increase the number of these partnerships. Likewise, these relationships could be optimized through increased and formalized communications between the MCP and plantations, continued monitoring and evaluation of progress, and the exploration of different types of participation by the private sector. Quantification of effectiveness of these partnerships requires an analysis of epidemiology on plantation sites and in nearby communities, and if this is done, it will assist the MCP to better target its resources.

The experience of Sabah provides an example of how private industries may support national and regional malaria elimination efforts. However, it is yet to be seen whether large-scale involvement of the private sector in regional malaria control, either through voluntary action or a legal framework, may better target and access at-risk groups to make regional elimination achievable.

## Abbreviations

IRS: Indoor residual spraying; ITN: Insecticide-treated net; MBS: Mass blood survey; MCP: Malaria Control Programme.

## Competing interests

The authors declare that they have no competing interests.

## Authors’ contributions

The text of this paper was drafted by KCS, CR, JJ, YR, CSG, and RG. All authors provided input on the paper development and focus. CR, JJ and YR provided guidance during in-country data collection and analysis. KCS collected and analysed data, with the support of CR, JJ and YR. CSG provided support during study development, data collection and analysis. All authors took part in the review, preparation, and final approval of the manuscript.
